# Technology usage and glycaemic outcomes in a single tertiary centre with an ethnically diverse and socioeconomically deprived cohort of children with type 1 diabetes mellitus

**DOI:** 10.3389/fcdhc.2024.1417287

**Published:** 2025-01-09

**Authors:** India Dickinson, Ankita Gupta, Gar Mun Lau, Pranav Viswanath Iyer, John Stuart Pemberton, Suma Uday

**Affiliations:** ^1^ School of Medical and Dental Sciences, University of Birmingham, Birmingham, United Kingdom; ^2^ Department of Endocrinology and Diabetes, Birmingham Women’s and Children’s NHS Foundation Trust, Birmingham, United Kingdom; ^3^ Department of Metabolism and Systems Science, College of Medical and Dental Sciences, University of Birmingham, Birmingham, United Kingdom

**Keywords:** type 1 diabetes, inequity, continuous subcutaneous insulin infusion, continuous glucose monitor (CGM), social deprivation, ethnic minorites, glycated haemoglobin

## Abstract

**Background:**

The UK National Paediatric Diabetes Audit (NPDA) data reports disparities in Haemoglobin A1c (HbA1c) levels among children and young people (CYP) with Type 1 Diabetes (T1D), with higher levels in those of Black ethnic background and lower socioeconomic status who have less access to technology. We investigate HbA1c differences in a T1D cohort with higher than national average technology uptake where > 60% come from an ethnic minority and/or socioeconomically deprived population.

**Design & methods:**

Retrospective cross-sectional study investigating the influence of demographic factors, technology use, and socioeconomic status (SES) on glycaemic outcomes. The study population was 222 CYP with T1D who attended the diabetes clinic in 2022 at a single tertiary paediatric diabetes centre.

**Results:**

Among 222 CYP, 60% were of ethnic minority (Asian, Black, Mixed and Other were 32%, 12%, 6% and 10% respectively) and 40% of white heritage. 94% used Continuous Glucose Monitoring (CGM) and 60% used Continuous Subcutaneous Insulin Infusion (CSII) via open or closed loop. 6% used Self-Monitoring of Blood Glucose (SMBG) and Multiple Daily Injections (MDI), 34% used CGM and MDI, 38% used CGM and CSII and 22% used Hybrid Closed-Loop (HCL) systems. Significant differences in HbA1c across therapy groups (p < 0.001) was noted with lowest HbA1c in HCL group (55 mmol/mol; p <0.001). Despite adjusting for therapy type, the Black group had higher HbA1c than their white and Asian counterparts (p<0.001). CYP from the most deprived tertile had significantly higher HbA1c levels (p < 0.001) but the difference was not sustained after adjusting for therapy type.

**Conclusion:**

Advanced diabetes technologies improve glycaemic control. Whilst equalising technology access mitigates socioeconomic disparities in HbA1c, CYP from Black ethnic background continue to display a higher HbA1c. The study underscores the necessity of fair technology distribution and further research into elevated HbA1c levels among Black CYP using advanced diabetes technology.

## Introduction

There are over 30,000 children and young people (CYP) in England and Wales with Type 1 Diabetes Mellitus (T1D) and the prevalence worldwide is reported to be substantially growing (1, 2). The assessment of glycaemic status, crucial for management, relies on haemoglobin A1c (HbA1c), a surrogate measure of 90-120 day mean blood glucose (MBG) (3). Effective glycaemic control, typically defined as a HbA1c < 48mmol/mol, is vital in improving the quality of life in CYP with T1D (4). The Diabetes Control and Complications Trial (DCCT) and its follow-up, the Epidemiology of Diabetes Interventions and Complications (EDIC) study, demonstrated that intensive therapy aimed at lowering HbA1c levels significantly reduces the risk of both early-stage and advanced complications in T1D in a dose-response manner (5). CYP of ethnic minority backgrounds and of lower socioeconomic status (SES) exhibit higher HbA1c levels than their counterparts in the UK National Paediatric Diabetes Audit (NPDA) data (6) and large international data sets (7).

The UK 2021/22 NPDA data reported a mean HbA1c of 63 mmol/mol for CYP with T1D from white ethnic background compared to 71 mmol/mol for their counterparts of Black ethnic background (8). Similarly, SES plays a significant role, with CYP from the most deprived quintile exhibiting a mean HbA1c of 68 mmol/mol, compared to 60 mmol/mol for CYP in the least deprived quintile (8). Complications caused by high glucose levels and negative health outcomes for CYP from Black ethnic origin are significantly influenced by the intersection of racial disparities and SES (9, 10).

The NPDA reported a 4 mmol/mol lower HbA1c in CYP using Continuous Subcutaneous Insulin Infusion (CSII) compared to those using Multiple Daily Injections (MDI) (8). However, ethnic disparities remain with only 29% of CYP from Black background using CSII compared to 42% of white CYP. Similarly, only 34% of CYP from the most deprived quintile used CSII compared to 45% from the least deprived quintile (8). The NPDA also revealed that individuals using continuous glucose monitoring (CGM) had lower HbA1c levels compared to those using self-monitoring blood glucose (SMBG) (8). However, it also found that access to CGM was disproportionately limited among ethnic minorities and socially disadvantaged groups (8).

This disparity in technology access among ethnic minorities and socially disadvantaged groups is also evident in studies from other countries, including Germany (11, 12) and the United States (7, 13).

Given that the proportion of CYP from ethnic minority (23%) and those from the lowest SES quintile (24%) in the national data (8) is low we aimed to evaluated technology uptake and glycaemic variability in our predominantly (>60%) ethnic minority and deprived cohort with higher than national average CGM uptake. Our aims were to compare technology usage at our centre, in different ethnic and socio-economic groups, to national data and to investigate the relationship between therapy type and glycaemic control among CYP with T1D in reference to ethnicity and SES.

## Methods

### Study design

A retrospective analysis of cross-sectional data collected from CYP with T1D attending the Diabetes Clinic at Birmingham Women’s and Children’s Hospital (BWCH) from 1st January 2022 to 31st December 2022.

### Study population

All CYP, aged 1-16 years, with T1D attending the diabetes clinic in 2022 at our centre, irrespective of current HbA1c value, were included. Within our centre, 60% of the cohort come from the most deprived socioeconomic quintile, and over 60% belong to ethnic minority groups. We provide care for around 300 CYP with T1D supported by an equivalent full-time staff comprising two consultant diabetologists, five paediatric diabetes nurses, two paediatric diabetes dietitians, one social worker, one family support/youth worker, and one psychologist.

Exclusion: CYP with less than two years diabetes duration (honeymoon) or changing insulin delivery method in 2022 were excluded to prevent confounding of HbA1c and technology relationships.

### Data collection

#### Demographics

Data was gathered from the online diabetes management system, TWINKLE. Data collected included age, gender, ethnic group, the need for an interpreter and postcode. Socioeconomic status (SES) was determined by Index of Multiple deprivation (IMD) which was computed using postcode (14). The IMD is the official measure of relative deprivation in England and follows an established methodological framework in broadly defining deprivation to encompass a wide range of an individual’s living conditions. The English Indices of Deprivation is based on 37 distinct indicators across seven domains, with the IMD scale ranging from 1 (most deprived) to 32,844 (least deprived) (14). The seven domains include: income, employment, health deprivation and disability, education and skills training, crime, barriers to housing and services; and living environment. Self-reported ethnicity according to the UK Census standards was classified as White, Asian, Black, Mixed or Other (including “not stated”) (15).

#### Therapy type

Insulin therapy and glucose testing method were obtained from the database and verified by the technology lead (JP). Insulin therapy was defined as Multiple Daily Injections (MDI), Continuous Subcutaneous Insulin Infusion (CSII) or Hybrid Closed Loop (HCL). Glucose testing was defined as Self-Monitoring Blood Glucose (SMBG) or Continuous Glucose Monitoring (CGM). In CGM+CSII cohort there was no communication between the glucose monitor and the insulin pump whereas in HCL cohort the CGM and insulin pump communicate to automatically adjust the insulin delivered through the pump in response to the blood glucose.

#### Glycaemic outcome data

HbA1c (mmol/mol) was recorded at three monthly clinic visits by point-of-care Siemens’ DCA Vantage (16). The mean annual HbA1c was calculated using the two to four most recent HbA1c values.

### Statistical analysis

All analyses were carried out using SPSS v29.0, with statistical significance set at p<0.05.

Individuals from Mixed (n=14) and Other (n=22, of which n=12 were reported as “not stated”) ethnic groups were excluded due to small sample sizes.

#### Continuous variable analysis

For therapy, ethnic and SES groups normality was assessed using the Shapiro-Wilk Test. Non-normally distributed variables are reported as median and interquartile range (IQR, 25^th^ to 75^th^). Between group differences were evaluated using the Independent Kruskal-Wallis test. *Post-hoc* pairwise comparisons, adjusted for multiple comparisons, were conducted to identify specific group variations. *Post-hoc* power calculations were performed using G*Power (17) to assess the risk of making type 2 errors.

#### Categorical variable analysis

The Chi-Square Test for Independence was used to investigate potential associations between demographic characteristics, technology utilisation and HBA1c for each of the therapy, ethnic and SES (IMD scores were categorised into tertiles) groups.

#### Secondary analysis

To explore the impact of excluding specific demographic groups, a secondary analysis was performed. In this instance, the Black ethnic group (n=26) was excluded, and analyses were conducted following the same procedures outlined above.

### Ethics

Data presented here is a secondary analysis of data that is routinely gathered and submitted to NPDA annually. The project was also registered with the BWCH audit committee (CARMS-31489).

## Results

The inclusion criteria were met by 222 (n = 116, 52% male) CYP with T1D (excluded: n = 40 diagnosed within 2 years, and n = 23 changed therapy type in 2022). The median age of the cohort was 13 (IQR: 11, 15) years and median annual HbA1c was 59 (IQR: 53, 67) mmol/mol.

### Comparison to national technology uptake based on ethnicity and SES

**Figure 1:**
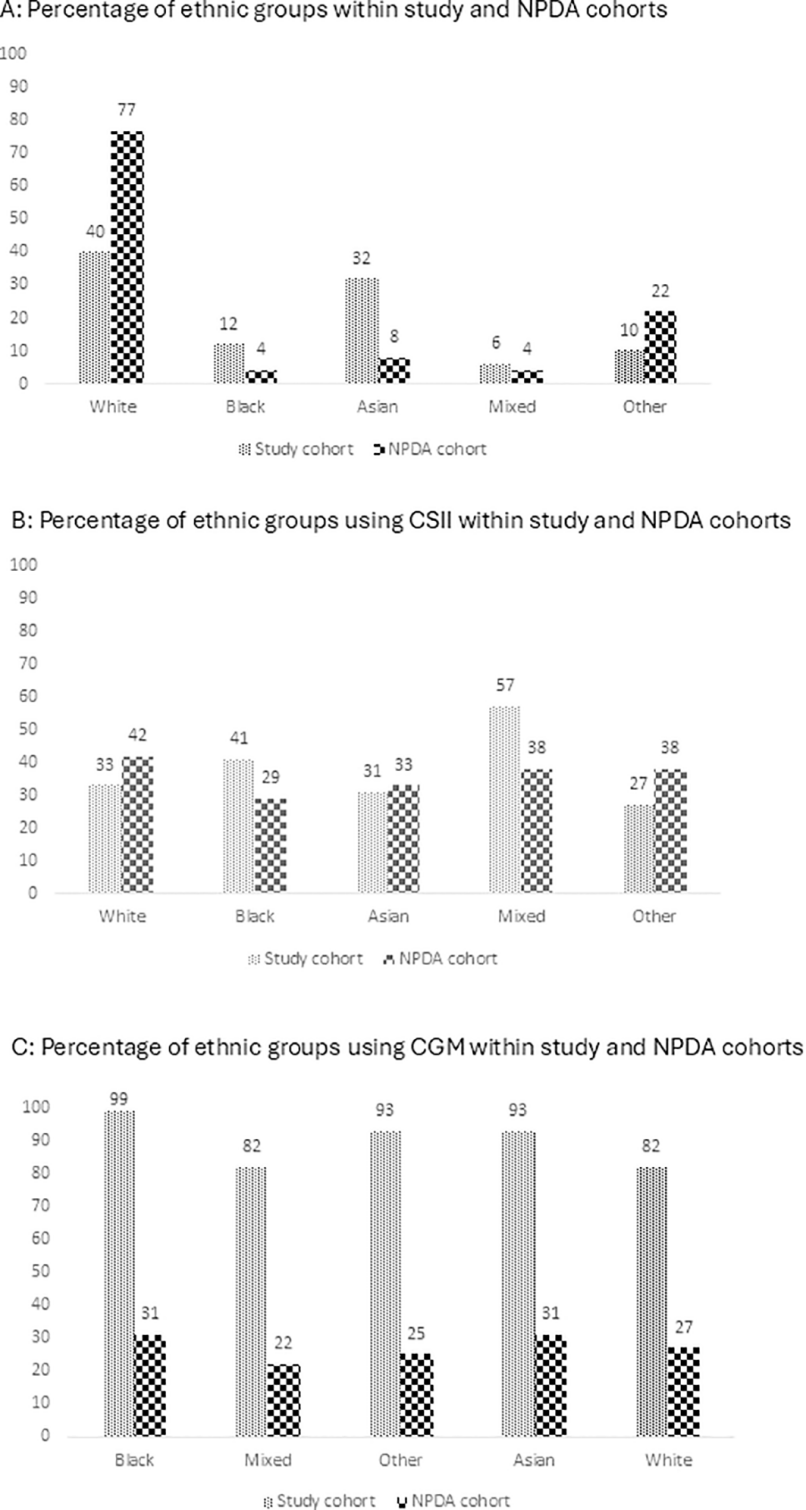
Access to advanced technology by ethnic group for the study group and NPDA cohorts in 2021/2022: **(A)** Ethnic Group. **(B)** CSII. **(C)** CGM CSII, Continuous Subcutaneous Insulin Infusion; CGM, Continuous Glucose Monitoring.

The study cohort were predominantly from minority ethnic backgrounds, with CYP from Asian, Black, Mixed and Other backgrounds accounting for 32% (n = 71/222), 12% (n = 26/222), 6% (n = 14/222), and 10% (n = 22/222) of the cohort respectively, with the remaining 40% (n = 89/222) being of white heritage. In contrast, the national dataset constitutes 77% white ethnic background, 22% reporting as Other ethnic origins, and less than 10% reporting as Asian, Black and Mixed heritage (**Figure 1A**). CSII provision across ethnic groups was similar in the study group and national data set, ranging from 29-57% (**Figure 1B**). However, access to CGM in our cohort was much higher, at 80% of participants across all ethnic groups accessing CGM, compared to 29-42% nationally (**Figure 1C**).

**Figure 2:**
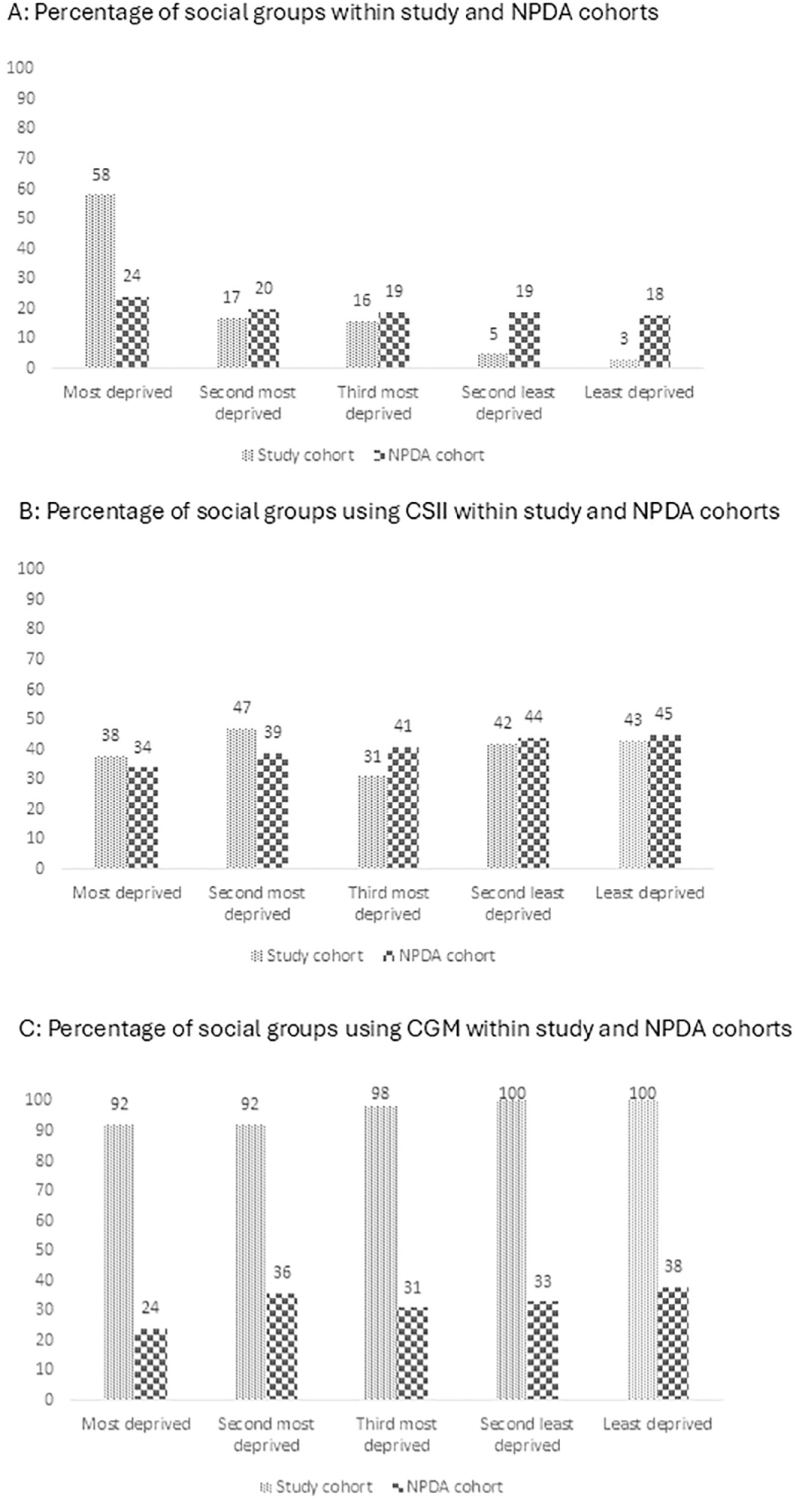
Access to Advanced Technology by Socioeconomic Status (Index of Multiple Deprivation Quintiles) for the study group and NPDA cohorts in 2021/2022: **(A)** Social group. **(B)** CSII. **(C)** CGM CSII, Continuous Subcutaneous Insulin Infusion; CGM, Continuous Glucose Monitoring.

The majority of CYP in our cohort resided in areas of high deprivation, with 59% (130/222) living in the most deprived areas and 17% (37/222) in the second most deprived area. 16% (36 out of 222), 5% (12/222) and 3% (7/222) were from the third most deprived, second least deprived and least deprived areas respectively. This distribution is in contrast with national data which is more evenly spread across deprivation quintiles, with each tier representing 18-24% of the population (**Figure 2A**). When examining access to CSII, the uptake in our cohort is closely aligned with national average, with both showing a range of 34-45% across different socioeconomic groups (**Figure 2B**). However, a significant discrepancy is observed in the adoption of CGM systems; more than 90% of participants across all socioeconomic strata in our cohort had access to CGM, in contrast to 24-38% uptake in the national dataset (**Figure 2C**).

### Therapy group analysis

**Figure 3:**
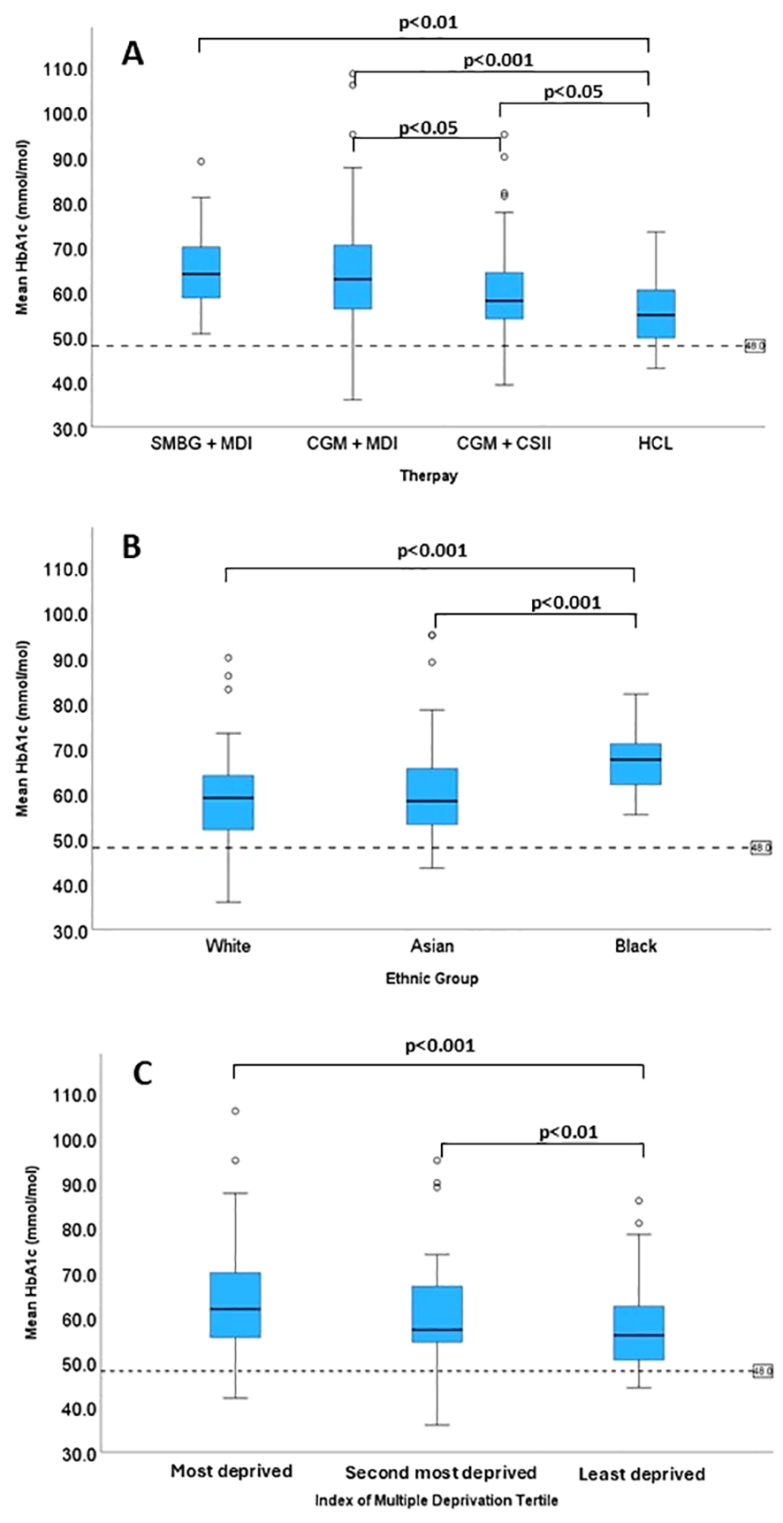
The pairwise comparison for **(A)** Therapy type, **(B)** Ethnic group and **(C)** Socioeconomic status.

Of the 222 CYP with T1D, 6% (n = 14/222) were using SMBG + MDI, 34% (n = 75/222) used CGM + MDI, 38% (n = 85/222) used CGM + CSII, and 22% (n = 48/222) used HCL. There were significant differences in HbA1c levels (p<0.001) across therapy groups (**Table 1**). CYP using HCL therapy had a median HbA1c of 55 (IQR: 50, 61) mmol/mol which was significantly lower than all the other groups (**Figure 3A**). CYP using CSII+CGM therapy had a HbA1c of 58 (IQR: 54, 65) mmol/mol which was significantly lower (p<0.05) than CGM + MDI therapy group who had a HbA1c of 63 (IQR: 56, 71) mmol/mol (**Figure 3A**). CYP using SMBG + MDI had the highest HbA1c, which was 64 (IQR: 58, 71) mmol/mol. There was a significant difference in IMD scores (p<0.001) across therapy groups (**Table 2**) with the HCL group having a significantly higher IMD (least deprived) score compared to all other therapy groups (p<0.01), whilst no other between group differences were observed. Interpreter requirement was different across the therapy groups (p<0.001), with the SMBG + MDI (36%) and CGM + MDI (17%) groups having the greatest representation. Ethnic group differences across therapy types did not reach statistical significance (p=0.077) despite CYP from white ethnic backgrounds making up 57% of the HCL group and only 7% of the SMBG + MDI group (**Table 1**).

**Table 1:** Demographics and clinical outcomes of different technology users.

	All (n=222)		SMBG + MDI (n=14)		CGM + MDI (n=75)		CGM + CSII (n=85)		*HCL (n=48)*		
	n	Median (IQR) Percentage	n	Median (IQR) Percentage	n	Median (IQR) Percentage	n	Median (IQR) Percentage	n	Median (IQR) Percentage	p value (power)
***Gender***											0.069^b^
Male	116	52%	9	64%	45	60%	35	41%	27	56%	
Female	106	48%	5	36%	30	40%	50	59%	21	44%	
Age (years)	222	13 (11, 15)	14	15 (13, 17)	75	13 (11, 15)	85	13 (11, 16)	48	13 (12, 15)	0.293^a^
Interpreter required	24	11%	5	36%	13	17%	5	6%	1	2%	<0.001^b^
Mean HbA1c (mmol/mol) (%)	222	59 (53, 67) 7.5 (7.0, 8.3)	14	64 (58, 71) 8.0 (7.5, 8.6)	75	63 (56, 71) 7.9 (7.3, 8.6)	85	58 (54, 65) 7.5 (7.1, 8.1)	48	55 (50, 61) (7.2, 7.7)	<0.001^a^ (98%)^c^

a Independent-Samples Krusksal-Wallis Test.

b Chi-Square of Independent-Square.

c *Post-hoc* power calculation using a 6 mmol/mol difference between therapy types, using a standard deviation of 10 mmol/L.

SMBG, Self-Monitoring Blood Glucose; MDI, Multiple Daily Injections; CGM, Continuous Glucose Monitoring; CSII, Continuous Subcutaneous Insulin Infusion; HCL, Hybrid Closed Loop.

### Ethnic group analysis

Of the 186 CYP meeting inclusion for analysis, 48% (n = 89/186) were white, 38% (n = 71/186) of Asian descent and 14% (n = 26/186) from Black ethnic backgrounds (**Table 2**). Of the CYP from white ethnic backgrounds, 1% (n = 1/89) used SMBG + MDI, 35% (n = 31/89) used CGM + MDI, 34% (n = 30/89) used CGM + CSII and 30% (n = 27/89) used HCL. Of the CYP from Asian ethnic backgrounds, 6% (n = 4/71) used SMBG + MDI, 31% (n = 22/71) used CGM + MDI, 44% (n = 31/71) used CGM + CSII, and 20% (n = 14/71) used HCL. In contrast, among CYP from Black ethnic background, 12% (n = 3/26) used SMBG + MDI, 35% (n = 9/26) used CGM + MDI, 42% (n = 11/26) used CGM + CSII and 12% (n = 3/26) used HCL.

**Table 2:** HbA1c based on technology use in different ethnic and socio-economic groups.

	All (n=186)		SMBG + MDI (n=8)		CGM + MDI (n=62)		CGM + CSII (n=72)		HCL (n=44)		
*Ethnic Group*	n (%) of ethnic group	Median HbA1c (IQR) mmol/mol %	n (%) of ethnic group	Median HbA1c (IQR) mmol/mol %	n (%) of ethnic group	Median HbA1c (IQR) mmol/mol %	n (%) of ethnic group	Median HbA1c (IQR) mmol/mol %	n (%) of ethnic group	Median HbA1c (IQR) mmol/mol %	
White	89 (100%)	59 (52, 64) 7.5 (6.9, 8.0)	1 (1%)	73 8.8	31 (35%)	61 (53, 69) 7.7 (7.0, 8.5)	30 (34%)	58 (54, 63) 7.7 (7.1, 7.9)	27 (30%)	55 (48, 61) 7.2 (6.5, 7.7)	
Asian	71 (100%)	58 (53, 66) 7.7 (7.0, 8.2)	4 (6%)	61 (53, 82) 7.7 (7.0, 9.7)	22 (31%)	63 (55, 70) 7.9 (7.2, 8.6)	31 (43%)	58 (54, 65) 7.5 (7.1, 8.1)	14 (20%)	56 (52, 59) 7.3 (6.9, 7.5)	
Black	26 (100%)	68 (62, 72) 8.4 (7.8, 8.7)	3 (12%)	68 (62, 74) 8.4 (7.8, 8.8)	9 (35%)	67 (58, 77) 8.3 (7.5, 9.2)	11 (42%)	67 (63, 71) 8.3 (7.9, 8.6)	3 (11%)	68 (68,68) 8.4 (8.4, 8.4)	
p-value Power		**<0.001^a^** **67% ^b^**		0.757^a^ 6%^b^		0.373^a^ 20%^b^		**0.002^a^** **30% ^b^**		**0.04^a^** **17% ^b^**	
	All (n=222)		SMBG + MDI (n=14)		CGM + MDI (n=75)		CGM + CSII (n=85)		HCL (n=48)		
Tertiles by IMD (median)	n (%) of tertile	Median HbA1c (IQR) mmol/mol %	n (%) of tertile	Median HbA1c (IQR) mmol/mol %	n (%) of tertile	Median HbA1c (IQR) mmol/mol %	n (%) of tertile	Median HbA1c (IQR) mmol/mol %	n (%) of tertile		Median HbA1c (IQR) mmol/mol %
1: 1188	74 (100%)	62 (55, 70) 7.8 (7.2, 8.6)	6 (8%)	60 (52, 68) 7.6 (6.9, 8.4)	34 (46%)	68 (58, 73) 8.4 (7.5, 8.8)	28 (38%)	60 (55, 68) 7.6 (7.2, 8.4)	6 (8%)		59 (43, 68) 7.5 (6.1, 8.4)
2: 4022	74 (100%)	60 (54, 67) 7.6 (7.1, 8.3)	6 (8%)	65 (61, 77) 8.1 (7.7, 9.2)	21 (28%)	62 (59, 67) 7.8 (7.5, 8.3)	30 (41%)	58 (54, 65) 7.5 (7.1, 8.1)	17 (23%)		60 (52, 68) 7.6 (6.9, 8.4)
3: 14354	74 (100%)	56 (50, 63) 7.3 (6.7, 7.9)	2 (3%)	70 (58, 81) 8.6 (7.5, 9.6)	20 (27%)	61 (51, 65) 7.7 (6.8, 8.1)	27 (36%)	57 (52, 63) 7.4 (6.9, 7.9)	25 (34%)		53 (48, 58) 7.0 (6.5. 7.5)
p-value Power		**<0.001^a^** **91%^b^**		0.432^a^ 8%^b^		0.144^a^ 25%^b^		0.235^a^ 50%^b^			0.055^a^ 9%^b^

^a^Independent-Samples Krusksal-Wallis Test.

^b^ *Post-hoc* power calculation using a 6 mmol/mol difference between groups, using a standard deviation of 10 mmol/L.

SMBG, Self-Monitoring Blood Glucose; MDI, Multiple Daily Injections; CGM, Continuous Glucose Monitoring; CSII, Continuous Subcutaneous Insulin Infusion; HCL, Hybrid Closed Loop.

Statistically significant values are indicated in bold.

With all therapy groups included, there was a significant difference in HbA1c between ethnic groups (**Table 2**) (p<0.001). CYP from Black ethnic background had a significantly higher HbA1c [68 (IQR: 62, 72) mmol/mol] compared to CYP from white background [59 (IQR: 52, 64) mmol/mol, p<0.001], and CYP from Asian background [58 (IQR: 53, 66) mmol/mol, (p<0.001)] (**Figure 3B**). The same pattern was observed for the CGM + CSII (p = 0.002) and HCL therapy groups (p = 0.04) (**Table 2**). For CGM + CSII (p<0.01) and HCL (p<0.05), HbA1c levels favoured CYP from white and Asian background when compared to CYP of Black ethnic background. The between groups differences for individual therapies were found under the condition of having no more than 30% statistical power (**Table 2**).

### Socioeconomic status analysis

The 222 CYP were grouped into tertiles based on IMD scores. There were 74 CYP in each tertile. The first tertile (most deprived) had a median IMD of 1188 (IQR: 824, 1458), the second was 4022 (IQR: 3011, 6209) and third (least deprived) was 14354 (IQR: 12907, 19910) with statistically significant difference across the groups (**Table 2**) (p<0.001). In the first tertile, 8% (n = 6/74) used SMBG + MDI, 46% (n = 34/74) used CGM + MDI, 38% (n = 28/74) used CGM + CSII, and 8% (n = 6/74) used HCL. In the second tertile 8% (n = 6/74) used SMBG + MDI, 28% (n = 21/74) used CGM + MDI, 41% (n = 30/74) used CGM + CSII, and 23% (n = 17/74) used HCL. In contrast, in CYP from third tertile 3% (n = 2/74) used SMBG + MDI, 27% (n = 20/74) used CGM + MDI, 36% (n = 27/74) used CGM + CSII, and 34% (n = 25/74) used HCL.

There was a significant difference in HbA1c between different tertiles (p<0.001) (**Table 2**). CYP from the first tertile had a significantly higher (p<0.001) HbA1c of 68 (IQR: 62, 72) mmol/mol when compared to CYP from the third tertile of 56 (IQR: 50, 63) mmol/mol (**Figure 3C**). CYP from the second tertile had a HbA1c of 60 (IQR: 54, 67) mmol/mol which was significantly higher than the third tertile (p<0.01), yet not significantly different from the first tertile (**Figure 3C**). No differences were found between tertiles in HbA1c levels for individual therapy types. However, there was no more than 50% statistical power which risks type 2 errors and must be considered upon interpretation. A secondary analysis was performed due to potential confounding effect of ethnicity and SES with 67% of the total CYP from Black heritage residing in the most deprived tertile and only 3% residing in the least deprived tertile. Upon excluding the 26 CYP from a Black heritage, almost identical results to the primary analyses were produced. However, caution must be applied due to less than 50% statistical power for the between IMD group analysis for the different therapy types.

## Discussion

Our study confirms that advanced diabetes technology offers better glycaemic control which is reflected in lower HbA1c levels associated with advanced technology usage. However, such technologies are not equally accessible to all demographic groups, indicating biases in distribution or barriers to uptake. Equalising technology access may reduce disparities attributed to SES, nonetheless CYP from Black ethnic background continue to exhibit higher HbA1c levels, despite advanced technology uptake.

The lowest HbA1c were observed in CYP using HCL systems and CGM + CSII. The majority of CYP using HCL systems resided in less deprived areas, and infrequently required an interpreter. In contrast, a significant proportion of CYP using SMBG and MDI live in most deprived areas, and frequently required an interpreter. We have previously demonstrated that glycaemic control is poor in CYP with T1D requiring interpreters as the presence of language barrier poses a significant hurdle to successfully educating CYP and their families (18). These CYP and their families should be provided with tailored support, for instance, diabetes specific training for interpreters and exploring other multi-dimensional factors contributing to poor glycaemic control (18). The low percentage of technology use in certain groups could be multi-factorial. A large German registry cohort of 29,284 CYP with T1D aged <20 years reported that the use of continuous glucose monitoring systems (CGMS) decreased from 6.3 to 3.4% in the least to the most deprived quintile (11). Over 50% of CYP using HCL are from white ethnic backgrounds, compared to only 7% using SMBG and MDIReal-world data from 13,351 German adults with T1D indicated that higher age, male gender, and migration background were associated with lower use of diabetes technology (12). Additionally, healthcare professional surveys show bias in offering advanced technology to CYP with T1D due to beliefs regarding the level of parental education and language proficiency required for technology use (19). The potential barriers from healthcare professionals may include cost and time constraints, as onboarding CYP requiring interpreters is more resource intensive (20). Reports of reduced uptake of advanced diabetes technologies amongst ethnic minority groups exist (21). The potential reasons for the lower adoption rate span across various levels, including societal, community, institutional, interpersonal, and individual factors (22). In our experience, the main barrier to technology uptake in our disadvantaged cohort include limited access to mobile devices and lack of digital skills, which hinder the ability to create online accounts and communicate with manufacturer customer services. However, the observation that only 23 individuals (10% of the cohort) transitioned from MDI to CSII therapy highlights a slow progression to more advanced treatment options. This may suggest therapeutic inertia or a lack of prioritisation for change, despite funding for CSII therapy not being a barrier within our service.

Despite access to effective technologies, our results identified that CYP from Black ethnic backgrounds exhibit higher HbA1c levels compared to their white and Asian counterparts, which would suggest they have higher average mean blood glucose. Interestingly, Christakis et al. (3) used comprehensive CGM glucose metrics to demonstrate a glucose-independent effect on HbA1c negatively affecting CYP of Black ethnicity. This study reinforces our findings by highlighting a potential additional glucose-independent effect in the Black ethnic population.

Encouragingly, equalising technology access seems to diminish HbA1c disparities across socio-economic groups. While disparities existed across all therapy types, they were not evident when advanced technology was accessible. This implies that the significant SES HbA1c disparities reported in England and Wales in 2021/22 could be mitigated through equitable technology access. Our analysis shows that all social tertiles achieve similar HbA1c outcomes when using HCL systems. However, the least deprived tertile had a 30% utilisation rate of HCL, compared to only 6% in the most deprived cohort, highlighting inequitable access. Achieving equity would require equal percentages of social groups utilising the most advanced technologies. While the overall usage rates may vary between centres, the gap between the most and least deprived groups should be minimal if equitable access is being achieved. Monitoring and auditing equitable access at the centre level is crucial to ensure that the national rollout of reimbursement for HCL systems is implemented fairly (23). Caution is necessary due to the low statistical power of the analysis and the complexity added by the majority of CYP from Black ethnic groups residing in most deprived areas. Future studies should aim to increase the sample size by pooling data from multiple centres/studies to enhance statistical power. Stratifying analysis by deprivation and ethnicity can capture distinct patterns in HbA1c amongst these subgroups. Employing multivariate statistical models or propensity scores will help control for this potential confounding. Despite the caution we apply to these implications, a large Australian study also confirms that HbA1c disparities across various socioeconomic strata is absent when matched for use of advanced technology (24).

Despite serving a population predominantly comprised of ethnic minority groups and experiencing significantly higher levels of social deprivation compared to national data, our centre reported a lower median HbA1c compared to the national average (61 vs. 62 mmol/mol). Additionally, after adjusting for factors such as ethnic group distribution and socioeconomic status, our centre was identified as a positive outlier for mean adjusted HbA1c in 2021/22. This positive performance may be attributed to the fact that over 80% of our diverse patient population utilises CGM, supported by a validated CGM education programme (25, 26).

Our retrospective analysis has limitations. The exclusion of individuals from Mixed and Other ethnic groups, due to small sample sizes, limits the ability of the study to capture true population diversity. The use of postcode-based SES indicators may oversimplify complex socioeconomic interplays through exclusion of parental education, occupation and specific household income. The small sample sizes in some subgroups may affect the reliability of subgroup analyses and depth of analysis. Furthermore, our results did not account for diabetes duration, age and baseline HbA1c. Our study was not designed to explore the factors, beyond ethnicity and SES, contributing to unequal technology access. The lack of CGM data restricted our ability to investigate glucose-independent effects accounting for any observed differences in HbA1c among ethnic groups. Future work exploring factors influencing equitable access to effective technologies should incorporate CGM metrics and hierarchical data modelling to better understand the complex relationships.

## Conclusion

Limited access to advanced diabetes technology disproportionately affects CYP with T1D from more deprived and ethnic minority backgrounds. As advanced diabetes technologies improve glycaemic control, healthcare professionals should be encouraged to inspect barriers to both uptake and offer of technology. Equalising technology access mitigates socioeconomic disparities in HbA1c but not ethnic disparities. CYP from Black ethnic background remain at risk of higher HbA1c suggesting a residual glucose-independent effect which necessitates further investigation.

## Data Availability

The raw data supporting the conclusions of this article will be made available by the authors, without undue reservation.
